# A systematic examination of international funding flows for Ebola virus and Zika virus outbreaks 2014–2019: donors, recipients and funding purposes

**DOI:** 10.1136/bmjgh-2020-003923

**Published:** 2021-04-13

**Authors:** Emily Jade Quirk, Adrian Gheorghe, Katharina Hauck

**Affiliations:** 1 School of Medicine, Imperial College London Faculty of Medicine, London, UK; 2 MRC Centre for Global Infectious Disease Analysis, Imperial College London School of Public Health, London, London, UK; 3 Abdul Latif Jameel Institute for Disease and Emergency Analytics, Imperial College London School of Public Health, London, UK

**Keywords:** health economics, health policy, public health, systematic review, other study design

## Abstract

**Introduction:**

There has been no systematic comparison of how the policy response to past infectious disease outbreaks and epidemics was funded. This study aims to collate and analyse funding for the Ebola epidemic and Zika outbreak between 2014 and 2019 in order to understand the shortcomings in funding reporting and suggest improvements.

**Methods:**

Data were collected via a literature review and analysis of financial reporting databases, including both amounts donated and received. Funding information from three financial databases was analysed: Institute of Health Metrics and Evaluation’s Development Assistance for Health database, the Georgetown Infectious Disease Atlas and the United Nations Financial Tracking Service. A systematic literature search strategy was devised and applied to seven databases: MEDLINE, EMBASE, HMIC, Global Health, Scopus, Web of Science and EconLit. Funding information was extracted from articles meeting the eligibility criteria and measures were taken to avoid double counting. Funding was collated, then amounts and purposes were compared within, and between, data sources.

**Results:**

Large differences between funding reported by different data sources, and variations in format and methodology, made it difficult to arrive at precise estimates of funding amounts and purpose. Total disbursements reported by the databases ranged from $2.5 to $3.2 billion for Ebola and $150–$180 million for Zika. Total funding reported in the literature is greater than reported in databases, suggesting that databases may either miss funding, or that literature sources overreport. Databases and literature disagreed on the main purpose of funding for socioeconomic recovery versus outbreak response. One of the few consistent findings across data sources and diseases is that the USA was the largest donor.

**Conclusion:**

Implementation of several recommendations would enable more effective mapping and deployment of outbreak funding for response activities relating to COVID-19 and future epidemics.

**Key questions**

**What is already known?**

- Low-income and middle-income countries are likely to require donations from development actors and high-income governments to manage the increased healthcare demand due to the COVID-19 pandemic.
- Outbreak funding contributions are documented in a variety of places, for example, specialised financial reporting databases, press releases, peer-reviewed literature.
- Existing fund tracking mechanisms have been described as insufficient and significant data challenges face researchers trying to collate funding information from many sources, but these difficulties are poorly characterised.

**What are the new findings?**

- A comprehensive and detailed list of donors and recipients of Ebola and Zika funding has been produced, highlighting a many discrepancies between international funding for outbreaks.
- Total literature-reported funding is greater than that in financial reporting databases and database-only totals varied by $2.5–$3.2 billion for Ebola (with $0.2 billion reported to have gone through the Ebola Multi-Partner Trust Fund) and $150–$180 million for Zika.
- Different data sources contained different fields (eg, funding descriptions), in different formats (eg, transaction date) and managed data differently (eg, used a different flow model).

**Key questions**

**What do the new findings imply?**

- Changes to financial reporting mechanisms are needed to obtain more reliable records of pandemic funding flows.
- Collating and maintaining a multitude of data sources hampers a clear, systematic overview of funding flows.
- Producers and users of funding data should formulate clear aims, weigh the advantages and disadvantages of each source and proceed cautiously with the analysis and interpretation of outbreak funding information.

## Introduction

The brunt of the impact of COVID-19 has initially been taken by high-income countries (HICs), in which the majority of deaths have occurred,^1^ but the virus has also affected low/middle-income countries (LMICs).^1–3^ Indirect impacts due to containment measures may be more stark in less developed countries^4–7^ and a significant lack of testing may be providing a false sense of security.^1 8 9^ LMICs are also less equipped to manage the patient with COVID-19 load—peak critical care demand in low-income countries is predicted to exceed capacity by 25.4 times^10^ and increased worldwide demand for personal protective equipment may reduce LMICs’ access^11^—creating the conditions in which health systems are susceptible to collapse.

Implementing recommended measures to curb virus transmission^3 5 10 12^ requires sustained funding. Most HIC governments are able to self-fund these mitigation measures, but LMICs often rely on international donations, loans and in-kind contributions (non-financial assistance like the deployment of personnel and supplies). The international community must now look outside their own borders as their case numbers and death tolls come down and assist less developed countries to prevent substantial mortality. This also reduces the risk of re-introduction of virus in countries that have it already under control—illustrating how outbreak funding can be framed as a ‘global public good’.^13^

Global health funding has been analysed extensively^14–16^ but relatively little is known about how international aid has been deployed in previous emergency outbreak situations, by whom, where and when. Existing financial tracking resources have previously been described as ‘not fit for purpose’ due to inconsistent reporting by donors^17^ and are even said to have exacerbated financing delays during the West Africa Ebola outbreak.^18^ Funding amounts recorded by different data sources vary,^17 19^ and researchers trying to combine funding information from these different sources face significant data challenges.^17^ This lack of effective fund stream monitoring and a (perceived) lack of accountability and fear of fraud^20^ may also hinder the international effort to raise donor support for the COVID-19 response.

The aim of this paper is to understand the shortcomings in funding reporting and monitoring for previous outbreaks, and suggest improvements, so the international community can put appropriate systems in place. We systematically analyse and compare funding contributions and amounts and funding purposes received by bilateral, non-governmental and private organisations, during the Ebola epidemic and Zika outbreaks between 2014 and 2019. A comprehensive assessment of funding flows is undertaken by combining a literature review with an analysis of financial information from multiple databases. Such a comparative analysis will inform how reporting should be organised to plan urgently needed international assistance in pandemic response. This will contribute to more efficient and faster funding of public health interventions and potentially save many lives.

## Methods

A two-pronged method was designed to capture international funding recorded in fund tracking databases as well as in published literature. Aid is referred to as a pledge, commitment or disbursements throughout to indicate how certain the funding is: pledges are announcements of intent^21^; commitments are ‘a firm obligation, expressed in writing and backed by the necessary funds’^22^ and a disbursement is an actual monetary transfer.^21^

### Analysis of financial data in databases

Development actors can report the aid they have donated or received to one of the multiple fund tracking databases. Three open-access databases containing financial data for Ebola and/or Zika were identified in February 2020, all of which contain donor contributions or funding obtained by recipients (table 1). The Development Assistance for Health (DAH) database contains Institute of Health Metrics and Evaluation (IHME) funding estimates, disaggregated in Ebola and Zika subsets. The Georgetown Infectious Disease Atlas (GIDA) has a financial tracking site recording global health security funding. The United Nations Office for the Coordination of Humanitarian Affairs (UN OCHA) manages the ‘Financial Tracking Service’ (FTS) in which actors report humanitarian funding. The FTS is the only database to include pledges and the DAH does not record commitments. The Organisation for Economic Co-operation and Development (OECD) Creditor Reporting System was considered for inclusion, but data would duplicate those in the IHME. The OECD system also does not disaggregate data by Ebola and Zika specifically, and it covers fewer funding sources than the IHME, such as private foundations.

**Table 1:** Financial reporting database characteristics including funding definition, sources of database information, relevant subsets, time periods for which data were available and perceived strengths, weaknesses and differentiating factors

Database	Funding definition	Data sources	Database range*	Data subsets	Common features	Differentiating features
Development Assistance for Health (DAH)^58^	Estimates of grants and loans for health projects in LMICs from bilateral agencies only	Project databases, financial statements, annual reports, internal revenue service forms and correspondence with agencies†^59^	1990–2018		Donor Recipient Disbursed amount Year of funding transfer‡ Downloadable online	Health focus area recorded (includes Ebola and Zika specific) Funding channel recorded (intermediate agency) Global burden of disease and World Bank region recorded Duplicate indicator field Preliminary estimate field Perceived strength: intermediate agency reported Perceived weakness: non-government funding is not disaggregated by donor except for the Bill and Melinda Gates Foundation
Georgetown Infectious Disease Atlas (GIDA) Global Health Security Tracking site^60^	Commitments and disbursements for global health security	Funding tracking initiatives, financial reports, media statements, press releases, directly from donors and funds†^61^	2014–2020			Committed amounts recorded Donor and recipient type Financial and in-kind support recorded and indicated by field Funding has an associated project name and description Related IHR core capacity is recorded Perceived strength: related IHR core capacity is recorded Perceived weakness: some funding lacked a description
Financial Tracking Service (FTS)^62^	Humanitarian funding (paid contributions and commitments)	Reported by participating actors	1980–2023	Incoming funds		Committed and pledged amounts recorded Funding source and destination described by cluster, response plan, objective, activity or project Exact date of funding transfer is recorded Usage year Perceived strength: includes exact dates of funding transfers Perceived weakness: relies on donor reporting alone
				Internal funding transfers		
				Outgoing funds		

*Database range refers to the years the database has been established; only data from 2014 to 2019 was used for this analysis.

†Full methods annex explaining participating actors and data sources on cited reference.

‡Some transactions have year ranges available instead of a single year for example, ‘2014–2016’.

IHR, International Health Regulations; LMICs, low/middle-income countries.

Funding information from 2014 to 2019 was identified, which includes the West Africa Ebola epidemic (2014–2015), three Democratic Republic of Congo Ebola outbreaks (2017, 2018 and 2018–ongoing) and the Zika outbreaks (2015–2017). In the GIDA database, the transaction year was often a multi-year interval (eg, 2014–2016), so any transactions including years outside the target time period (eg, 2013–2014) were excluded. For the DAH database, information was only available until 2018. Ebola and Zika funding information was already present as a separate field in the DAH database. The GIDA did not segregate funding by health focus, so the database was downloaded and searched manually in Excel to locate funding with ‘Ebola’ or ‘Zika’ in the project name or description; and new Ebola-specific and Zika-specific datasets were created. The FTS database allows funding sources and destinations to not only be a country or organisation, but also an emergency, response plan, organisation or project.^23^ Data were only able to be downloaded separately for pre-set fund groupings so transactions from the (pre-set) Ebola-related groupings, detailed in online supplemental table A1, were downloaded and combined into one dataset.

10.1136/bmjgh-2020-003923.supp1 Supplementary data

As the GIDA was the only database to contain funding descriptions, it was also the only database allowing the analysis of funding purposes. Transactions were classified as having one, or more, of five purposes: response, preparedness, socioeconomic recovery, research and unclassifiable. Online supplemental table A2 displays the classification criteria used. Further disaggregation of socioeconomic recovery funding was not possible, as GIDA funding descriptions did not contain enough detail to enable this.

In some cases, the DAH database reports only preliminary estimates—these were included in the analysis. The FTS separates fund transfers into data subsets (incoming funds, internal transfers between UN agencies and outgoing funds) to prevent double counting.^23 24^ We examined only incoming funds, which represent donations made to Ebola-related activities. Duplicates were removed within each dataset and funding information was summarised by subgroups, including funding destination, source and year.

Administrators of GIDA and DAH were contacted in March 2020 for specific clarifications on data availability and sources, but GIDA did not respond and DAH answered in May 2020, after the analysis had been completed.

### Analysis of financial data in the literature

Academic literature containing financial amounts donated or received for Ebola and Zika from 2014 to 2019 was sought. A systematic search strategy was devised and applied to seven databases: MEDLINE, EMBASE, HMIC, Global Health (all four searched via Ovid), Scopus, Web of Science and EconLit. A full list of search terms is available in online supplemental table A3.

Preferred Reporting Items for Systematic Reviews and Meta-Analyses (PRISMA) guidelines were followed in the screening and exclusion of articles.^25^ Search results were exported to EndNote reference management software, duplicates were removed, and titles and abstracts were screened for relevance to aims. Included full-text articles were then screened against eligibility criteria (online supplemental table A4) by two reviewers and disagreements were resolved through discussion. Articles containing Ebola or Zika funding for measures that respond to, prepare for or aid recovery for outbreaks were included, with no restrictions on study design or level of evidence. Non-English articles and articles with a study period pre-2014 were excluded.

Funding information was extracted from eligible articles, including transaction date, source of the financial data, the names of donors and recipients and the funding amount. The funding purpose was classified into one or more transaction purpose types (see online supplemental table A2). Outside financial databases, alternative words to ‘pledge’, ‘commitment’ and ‘disbursement’ are often used to describe the certainty of transactions, for example, ‘announcement’ or ‘provided’. When extracting financial data from the literature, transactions were assessed on a case-by-case basis and classed as a pledge, commitment or disbursement.

Two separate datasets were generated, one for donors and one for recipients, henceforth referred to as ‘Literature by donor’ and ‘Literature by recipient’. This was done by separating transactions into four different groups, depending on whether they contained donor, recipient or more general funding information:

1. Articles that calculated/quoted a value for the total pledged, committed or disbursed to Ebola or Zika by all actors in a certain timeframe.
  For example, the total amount disbursed towards the 2014/2015 Ebola outbreak was…
2. Amounts pledged, committed or disbursed from a named donor but with no recipient information.
  For example, the UK disbursed £10 million GBP to help fight Ebola.
3. Amounts received in pledges, commitments or disbursements by named recipient but with no donor information.
  For example, Guinea received US$5 million to help combat Ebola.
4. Amounts pledged, committed or disbursed from a named donor to named recipient.
  For example, the USA pledged US$1 million to the WHO Contingency Fund for Emergencies.

Type 4 transactions were manipulated into both donor and recipient forms and included within type 2 and 3 transaction groups. For example, China pledging $6 million to the World Food Programme would be included in the donor dataset as a pledge of $6 million to Ebola general and included in the recipient dataset as the World Food Programme receiving $6 million in pledges. Type 1 transactions were kept separate and compared only to each other. Total and actor-specific pledges, commitments and disbursements were calculated for the two datasets—online supplemental tables B1–B9 provide a full list of values and source articles.

Duplicate transactions with the exact same monetary value, purpose, donor or recipient and time frame within each dataset were excluded. Source articles were revisited to clarify possible overlaps or double counting. For example, one article stated that the USA had committed $5.4 billion^26^ while another publication gave a breakdown of this amount into budget appropriations^27^—in this case, only the information from the second article was included, as it contained more specific information about the funding destination.

Transactions with the same donor or recipient and purpose in an overlapping time frame were combined into a new transaction, with the value depicted as a range. This accounts for both the possibility that the transactions are actually the same funding and the possibility that they are different, preventing double counting as well as underestimation. For example, $163 million disbursed between January and October 2014,^19^ was combined with $249 million disbursed by the same donor between September 2014 and January 2015^28^ to make a collective disbursement of $249–$412 million between January 2014 and January 2015.

All financial values from the databases and literature were converted into constant 2019 US$, using conversion factors from a web-based tool^29^ that accounts for inflation and currency conversion. If the original transaction date was a range, the upper year was used for the original currency year.

### Patient and public involvement

It was not appropriate or possible to involve patients or the public in the design, or conduct, or reporting, or dissemination plans of our research.

## Results

The three databases were found to have several commonalities but a notable degree of variation in terms of the type of information recorded, data sources, relevant subsets, available time period and key fields for each database. These features and the perceived strengths and weaknesses are displayed in table 1.

Nine hundred and ninety articles were identified in the literature search, of which 352 were duplicates (PRISMA flow diagram^25^ in online supplemental figure C1). Two main types of publication were identified: articles with a primary aim of calculating funding totals; and articles quoting isolated numerical values alongside other information. There were also articles describing in-kind contributions from China^30–32^ and the USA.^27^ No information regarding loans (eg, from the International Monetary Fund) was documented. As few transactions from named donors to named recipients were obtained, analysis showing funding flows between exact actors was unachievable. In most literature sources there was insufficient information to determine definitively if separately quoted amounts represented the same transaction, therefore a large proportion of data is depicted as a range—particularly for Ebola funding.

### Ebola funding

**Figure 1:**
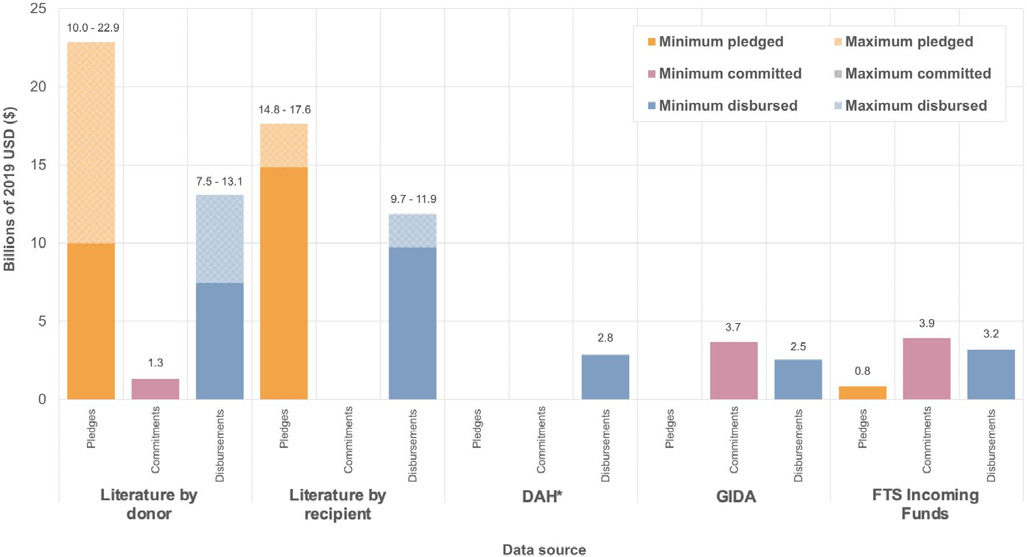
Total pledged, committed and disbursed to Ebola according to each data source. Minimum and maximum values are used to illustrate the discrepancies in reported amounts between different source articles. *DAH data only available from 2014 to 2018. DAH, Development Assistance for Health; FTS, Financial Tracking Service; GIDA, Georgetown Infectious Disease Atlas; Literature, articles containing Ebola and/or Zika funding donor contributions (‘by donor’) and received funding (‘by recipient’) from 2014 to 2019 identified in a search of MEDLINE, EMBASE, HMIC, Global Health, Scopus, Web of Science and EconLit.

Ebola funding information was extracted from 23 articles. From 2014 to 2019, total disbursements to Ebola ranged from $2.5 billion to $13.1 billion (figure 1) depending on the data source being considered.

Several articles calculated the total amounts pledged, committed or disbursed in total for Ebola outbreaks (online supplemental table C2). The total amount disbursed to the 2014–2015 Ebola outbreak according to a UN report^28^ was almost six times the total calculated by Grépin,^33^ despite including data from fewer months. The total amount pledged according to Glassman^19 34^ and Grépin^33^ were fairly similar ($2.6–$3.8 billion) but the UN report total was significantly higher, at over $9 billion.^28^ Two articles used the UN OCHA FTS to produce their totals^19 33^ and Glassman used data from UN OCHA, but it was not clear whether this was the FTS.^34^ Other data sources used press releases,^34^ information requests and the Ebola Multi-Partner Trust Fund (MPTF).^28^ Moss^35^ calculated the total amount provided by all donors for the DRC Ebola outbreak from August 2018 to December 2019 using a variety of publicly available information, mostly from OCHA and the WHO.

#### Individual actor funding

Eighty-two actors pledged funds to Ebola and 91 donors were documented to have actually disbursed funds, according to articles identified in the literature search. A full list of donors and their pledged, committed or contributed amount is available in online supplemental tables B1–B3. The USA is the top donor by a large margin according to the DAH and the literature (online supplemental figures C2 and C3). However, it appears some of this may be national spending, as according to the literature, US agencies and jurisdictions received 23%–36% of Ebola funding. The UK was the second highest donor according to the DAH but the third highest according to the literature, after the European Union.

Sierra Leone, Liberia and Guinea were the top direct recipient countries for Ebola for both data sources, but the literature reported that the UN, the WHO and non-governmental organisations received high amounts as well. The DRC received $0.8 million for Ebola according to the DAH database, though it is important to note that 2019 data is not included. The DRC also does not appear as a recipient in the literature as no articles recording the receipt of Ebola funding post-2015 were identified.

Furthermore, the DAH database and the literature reported $885 million and $679 million of Ebola funding was disbursed to unrecorded recipients respectively and DAH recorded substantial contributions by ‘Other’ donors and ‘Debt repayments’ for Ebola (online supplemental figure C2). Also, in the literature, some recipients were non-specific groups or types of recipient for example, ‘Other NGO’.

#### Funding purposes

**Figure 2:**
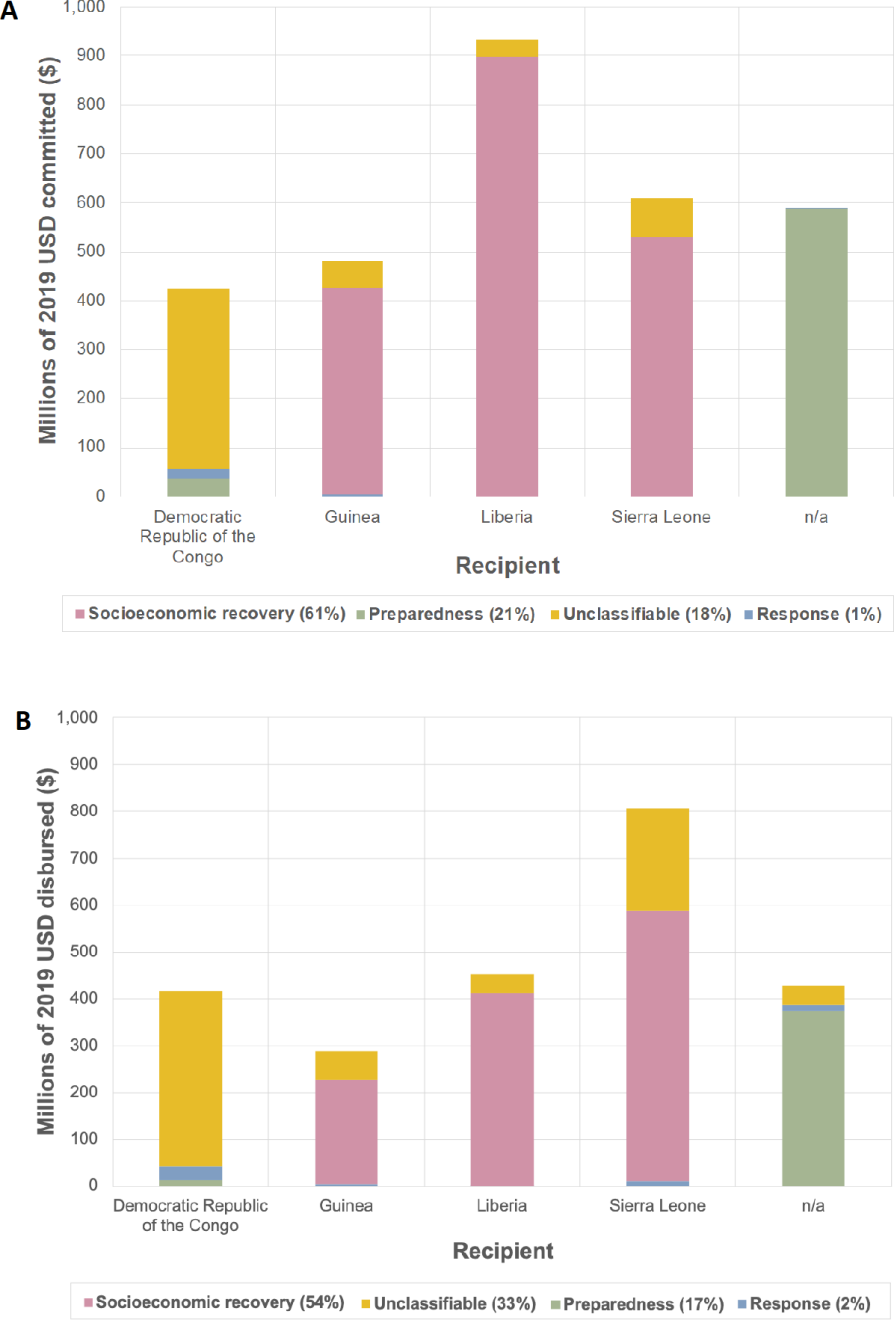
Proportions of funding reported by the Georgetown Infectious Disease Atlas (GIDA) received by the top 5 recipients for different purposes (a) Commitments (b) Disbursements. Overall percentage of funding for each purpose is stated in the legend.

Funding purposes recorded in the GIDA database varied by source and recipient (figure 2). The majority of funding received by Guinea, Liberia and Sierra Leone was earmarked for socioeconomic recovery—61% of commitments and 54% of disbursements. Seventeen per cent of funding disbursed was for preparedness, though almost all of this was disbursed to unnamed recipients, and little funding was designated for response. A third of all disbursed funding was ‘Unclassifiable’, most apparent in the DRC where unclassified funds constituted the majority received.

The proportion of Ebola funding reported for each purpose in the literature was different to the GIDA-reported purposes, with 97%–98% of ‘literature by donor’ disbursements designated for response and the remainder for research and preparedness. Response funding was for purposes such as building ‘isolated wards for patients with Ebola and buying of necessary protective equipment’.^36^

### Zika funding

**Figure 3:**
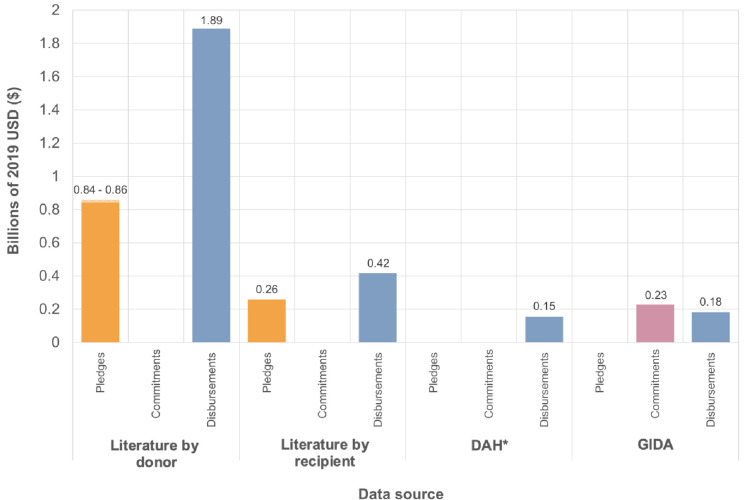
Total pledged, committed and disbursed to Zika according to each data source. Minimum and maximum values are used to illustrate the discrepancies in reported amounts between different source articles. *DAH data only available 2014-2018. DAH, Development Assistance for Health; GIDA, Georgetown Infectious Disease Atlas; Literature, articles containing Ebola and/or Zika funding donor contributions (“by donor”) and received funding (“by recipient”) from 2014-2019 identified in a search of MEDLINE, EMBASE, HMIC, Global Health, Scopus, Web of Science and EconLit.

Markedly less Zika outbreak funding information was obtained, with information extracted from only eight articles. Most transactions were from 2016, with one transaction documented in 2017 and there were no articles that calculated the total funding contributed to the 2015–2017 outbreak. Total disbursements to Zika ranged from $154 million to $1.9 billion (figure 3), depending on the data source.

#### Individual actor funding

Relatively few donors and recipients were recorded for Zika according to the literature with seven development actors pledging funds and four disbursing funds (a complete list can be found in online supplemental tables B4 and B5). Though there was a greater presence of research institutions and pharmaceutical companies compared with Ebola. The USA was the donor that contributed the most, however, according to the literature, a startling 95% of this was received by US agencies and jurisdictions.

A large portion of the funding did not have a named donor or recipient—92% of Zika funding was received by ‘Global’ and ‘Unallocated/Unspecified’ according to the DAH database (online supplemental figure C3).

#### Funding purposes

Analysis of specific funding purposes for Zika using GIDA data was unfeasible, as 39 of the 40 items of financial information were unclassifiable due to vague or lacking descriptions. The majority of Zika donor disbursements recorded in the literature were for response (66%) but a greater proportion of the funding was for Zika research (13%) and preparedness/response (21%).

### Pledges, commitments and disbursements

Funding commitments are not documented in the literature as much as pledges and disbursements—only $1.3 billion was recorded to have been committed by donors for Ebola and no Zika funding commitments were recorded at all (figures 1–3). Pledges were greater than disbursements for Ebola, according to the literature, and commitments were greater than disbursements according to the GIDA and the FTS (figure 1). This is the same for GIDA-recorded Zika funding, but the opposite for Zika literature transactions, with over $1 billion more disbursed than pledged (figure 3). Also, $1.5 billion more is recorded in the ‘literature by donor’ subset than the ‘literature by recipient’ subset for Zika. Although for Ebola, the amounts may be more similar, depending on whether one considers the minimum or maximum values for the two subsets.

### Comparing literature and database totals

In the literature, the types of donors reporting funding include government agencies, financial institutions, foundations, NGOs and UN agencies, whereas the DAH database mainly only includes bilateral government funding (online supplemental figures C2 and C3).

The amounts pledged, committed and disbursed differ by data source. For both diseases, the literature-reported totals were markedly higher than the database totals—the databases documented between $2.5 and $3.2 billion in disbursements for Ebola, whereas the literature recorded at least twice this (figure 1). Out of the databases, the FTS reported the most disbursements for Ebola ($3.2 billion), followed by the DAH and then the GIDA. Whereas, for Zika GIDA reported higher disbursements than DAH (figure 3).

This discrepancy is also seen on an individual actor level, with amount donated by actors according to the literature consistently greater than DAH-reported funding (online supplemental figures C2 and C3). The largest difference is seen for Ebola funding contributed by the USA, with between $1.6 and $5.7 billion more reported in the literature than the DAH database. Similarly, for Zika the literature-reported total amount donated by the USA is 10 times higher than the DAH database total. The amount received varies less, but still noticeably. Guinea received the least according to both data sources and Liberia received the most according to the literature, but Sierra Leone the most according to the DAH.

There were also discrepancies between the amount committed and disbursed for certain purposes (figure 2), for example, Liberia received less in disbursements than it did in commitments for socioeconomic recovery and preparedness. In the literature, a notable amount of literature funding was designated for more than one purpose, for example, $1.7–$2.6 billion was pledged for Ebola preparedness/response/socioeconomic recovery. No multi-purpose transactions were present in the GIDA data. There also appeared to be less received for certain purposes than was being pledged or donated by a donor, for example, there was nearly 60 times less funding for Zika research received ($4 million) than was donated ($251 million).

## Discussion

### Summary of findings

Reported total disbursements over 2014–2019 ranged from $2.5 to $13.1 billion for Ebola and between $154 million and $1.9 billion for Zika, depending on the data source. Considering only the databases, variability was less but still sizeable—$2.5–$3.2 billion for Ebola and $150–$180 million for Zika. The USA was consistently the top donor across multiple data sources, with the UK and the European Union also among the most generous contributors. The GIDA reports ‘socioeconomic recovery’ as the predominant funding purpose but response is more prominent in the literature. Significant discrepancies were identified in total amounts and types of information reported between, and within, different funding data sources, which makes it difficult to reach consensus on the nature of the overall funding flows.

Our findings align with current literature that well establishes the USA as the top donor for outbreaks^19 28 33 35^ and health generally.^16^ However, our observation that much of this funding is received by US agencies or jurisdictions, has only been previously noted once.^28^ The apparent unfulfillment of pledges and commitments with disbursements, found for Ebola, is aligned with previous findings,^19 33 34^ although the opposite was the case for Zika. This may be as donors are disbursing without first pledging, or that Zika funding pledges are not being recorded. High proportions of funding for response (79%) and research and development (3%) have been found previously,^28^ supporting our literature-reported findings. However, according to the GIDA, a much higher proportion of funding was for socioeconomic recovery or was unclassifiable. This was markedly different to previous findings, where only 18% of donor funding was designated for recovery and 3.6% recipient-reported expenditure was unclassifiable.^28^

This study is the first, to our knowledge, to not only collate funding information from multiple outbreaks using a literature review, but to report that the literature totals are greater than the databases’ and that totals also vary between databases. Discrepancies between data sources for a single donor may be as high as $5.7 billion, significantly more than cited in previous literature which states differences of only $100 million^17^ and $391 million.^19^ A possible explanation for these differences is double counting of funding disbursements from governments who use multilaterals as an intermediary—but it is hard to know the degree of impact of this on the results. This suggests the databases may represent an incomplete picture of the funding streams, that the literature sources tend to overreport, or a combination of the two. If one considers that additional databases and interactive dashboards tracking global health funding also exist,^37 38^ the disjointed and conflicting nature of outbreak fund tracking becomes increasingly apparent.

Monitoring funding is necessary to identify remaining funding requirements,^39^ ensure mutual accountability and to recognise the contributions of donors.^40^ COVID-19 response funding is being monitored by various institutions^41–46^ but these mechanisms may fall victim to the same shortcomings as Ebola and Zika fund tracking methods. Inconsistencies between fund reporting mechanisms may dissuade or delay donors from making contributions to the COVID-19 response, as there is little information on how infectious disease outbreaks are funded, let alone evidence-based guidance on how to allocate funds effectively. Furthermore, recent political decisions may significantly effect global health donations, particularly from the USA, who recently announced a termination of the global power’s relationship with the WHO^47^—this appears to have already taken effect, with only 4.2% of contributions to the COVID-19 appeal being from the USA.^42^ Their promises to reallocate tremendous amounts of funding to ‘other worldwide and deserving, urgent global public health needs’ exemplify that effective fund tracking has never been more important. Though in and of itself, data transparency will not resolve this problem fully—more analysis of funding impact would also be necessary.

#### Limitations

The few similarities between the format of the examined databases invite caution when interpreting the results. The funding date was given as a range of years, single year, month or exact date in different databases. Fields were also dissimilar, for example, GIDA has funding descriptions, DAH reports the intermediate agency that funding was transferred via and the FTS is the only database to record pledges. Database methodology also varied, with differing inclusion criteria, ways of accounting for duplicates and actors types that report, for example, funding transfers from the US government to the department of defence and human services do not appear in the FTS.^19^ These variances are also prevalent in the literature, for example, Ravi *et al*^48^ reported relevant information but not at the appropriate granularity. These differences in reporting style, also discussed elsewhere,^17^ mean there are many factors to consider when combining information and deciding which source’s values are ‘correct’. Nevertheless, they do provide reasons for the discrepancies between reported amount, and purpose, found in this study. We also examined the Ebola Response Multi-Partner Trust Fund database maintained by the United Nations Development Programme,^49^ but ultimately decided to exclude it from the analysis because the amounts were much smaller compared with the other three databases.

The novel approach taken by this study, combining financial values from articles identified in a literature review, also has limitations. Almost all articles identified by the literature search were editorials, journal blog posts or grey literature, meaning the level of evidence is weak. There was difficulty balancing between combining transactions into ranges, to prevent double counting and keeping transactions separate to provide an accurate picture of funding purposes. Furthermore, the literature totals are still likely to include duplicate funding, as intermediate holders of funding are counted as both donors and recipients. Additionally, much of the ‘literature by recipient’ funding information comes from donors, with disbursements not confirmed by recipients. So even though the literature appears to show more funding was donated than received, actually, donors are not reporting the destination of the funding. Due to the wording of the literature, it was also often difficult to concretely classify something as a pledge, commitment or disbursement, and determining a single purpose was difficult as funding descriptions were often vague, for example, ‘to address the Ebola outbreak’.^28^

### Recommendations

#### For organisations managing databases

Several modifications to financial tracking databases should be considered to enable more definitive, reliable overviews of funding flows. Standardising the format of information across the databases would mean that information from different reporting systems can be collated more effectively and efficiently. This could be done by increasing collaboration and communication between the organisations who manage these databases, as has begun to happen between the International Aid Transparency Initiative and the UN OCHA FTS.^50^ Data collection methods would not necessarily have to be the same, just highlighted explicitly so that any efforts to combine data from multiple sources are less susceptible to double counting. Existing donor reporting standards (eg, OECD reporting standards^51^) would also have to be considered when designing the standardised format.

Beneficial characteristics would be having pledges, commitments and disbursements (financial and in-kind) all recorded at least monthly but ideally in real time, enabling valuable comparisons with case numbers. Having a flexible reporting format, as the FTS does,^23^ to account for the reality that funding cannot always be reported in a generic donor to recipient format, may mean the funding picture is more complete. Detailed descriptions of funding purpose and the health focus should be included, as without this information it is more difficult for researchers and policymakers to determine how funds were used and whether funding fulfils its purpose. Additional features, such as a field highlighting duplicate transactions (as the DAH has) and separating funding into incoming funds, internal fund transfers and outgoing funds (as the FTS does) may prevent double counting.

#### For donors

Donors should continue to be urged to report their financial donations, as databases can only provide complete and accurate information if there is similarly accurate and diligent reporting. Recording responsibility should be placed to a greater extent on donors than on recipients, as disease-affected countries are often managing humanitarian crises alongside outbreaks,^35^ and their health systems are under immense pressure—in such circumstances, having a single database to report to, with clear, rehearsed reporting protocols could improve the quality of reported data. Also, developing countries often lack the resources to manage the large amounts of funding they receive from abroad.^19^ Donors may be averse to reporting due to the time lag between work completion and funding payments, preferring to report only commitments—but both pieces of information are required for a complete picture.^17^ Furthermore, researchers and database-managing organisations often categorise funding purpose subjectively or using string search terms,^52^ so if donors classify the intended use of funds themselves, the purpose will likely be more accurate.

#### For future research

Our findings warrant a future comprehensive comparison of all financial reporting databases for outbreaks. Comparisons of funding estimates, trends and methods have been already been made for reproductive, maternal and child health.^53^ Qualitative accounts from organisations managing databases would be valuable, as they may provide a more detailed insight into the methodology and format of databases as well as the perceived feasibility of changes and collaboration. Despite attempts to contact these organisations, this was not possible in this study. Determining the views of donors and recipients would further highlight the strengths and weaknesses of fund reporting mechanisms and may help determine reasons for missing data. Available studies have looked at general health system funding^54^ and its determinants,^55^ and it would be constructive to explore this in an outbreak context. Funding from other sectors related to outbreak response, such as food safety, animal health and the military, could also be investigated.^56 57^

### Conclusion

This study identified considerable differences in reported values and purposes of international funding for Ebola and Zika outbreaks. It also highlighted the challenges in constructing a comprehensive, high-resolution picture of global funding flows for outbreak preparedness, response and recovery. Standardisation of reporting database formats and in-depth comparison of methodology would enable reliable overviews of funding flows to be determined more easily. Increased communication and collaboration between the databases and further research to characterise the differences would help achieve this. These policy changes may facilitate more accurate and definitive mapping of the complex funding landscape, ultimately leading to better spending that could save lives and protect livelihoods from the COVID-19 pandemic and the inevitable future damaging epidemics.

## Data Availability

Data are available in a public, open access repository. All data relevant to the study are included in the article or uploaded as supplementary information. Data used in this study have been sourced from publicly available databases. The newly generated datasets underpinning the analysis are included in the supplementary material. Additional information will be provided by the corresponding author upon reasonable request.
