# Supplementary material for: A systematic examination of international funding flows for Ebola virus and Zika virus outbreaks 2014–2019: donors, recipients and funding purposes
Source: BMJ Glob Health. 2021 Apr 13;6(4):e003923. doi: 10.1136/bmjgh-2020-003923 (PMC8051378; doi:10.1136/bmjgh-2020-003923)
Supplement: Supplementary data [file bmjgh-2020-003923supp001.pdf]

A SYSTEMATIC EXAMINATION OF INTERNATIONAL FUNDING FLOWS FOR EBOLA VIRUS AND ZIKA VIRUS OUTBREAKS 2014 – 2019: DONORS, RECIPIENTS AND FUNDING PURPOSES

Supplementary Material

Supplement 1 – Methods

Table A1. Pre-set groupings downloaded and analysed from the United Nations Office for the Coordination of Humanitarian Affairs (UN OCHA) Financial Tracking Service (FTS).

**Grouping type**	**Groupings used**
Emergency	- DR Congo - Ebola Outbreak 2018 – 2019<br>- Ebola - WEST AFRICA - July 2015<br>- Ebola Virus Outbreak - WEST AFRICA - April 2014
Organisation	- Ebola Response Multi-Partner Trust Fund<br>- Global Ebola Response Coalition<br>- UN Mission for Ebola Emergency Response
Plan	- Ebola Virus Outbreak - Overview of Needs and Requirements (Inter-agency plan for Guinea, Liberia, Sierra Leone, Region) - October 2014 - June 2015
Project	- EBOLA-14/H/71120/122<br>- EBOLA-14/CSS/72256/561<br>- EBOLA-14/F/71114/561<br>- EBOLA-14/H/71122/99

Table A2. Funding purpose classification criteria.*

Purpose	Description
Response	Immediate or short-term activities often in the form of medical equipment, vaccines, medication, diagnostics and personnel. Humanitarian assistance and distribution of essentials such as food, water and shelter were also included. Staff training was included if it was short-term to deal with the current crisis.
Preparedness	Health system strengthening, improved surveillance, contributions to emergency funding pots like the WHO Contingency Fund for Emergencies, long-term staff training.
Socioeconomic recovery	To help rebuild damaged economies and long-term support to businesses and individuals.
Research	Immediate, short-term and long-term financial support to research institutions, laboratories and pharmaceutical companies to develop new treatments, vaccines or to increase the knowledge about the disease.
Unclassifiable	Non-English description, lacked a description or had an ambiguous description that meant a clear purpose could not be determined.

*This classification was only applicable for the Georgetown Infectious Disease Atlas funding database.

Table A3. Literature search terms applied to each database.

m.p. = key word in abstract, title, original title, broad terms, heading words, identifiers, cabicodes

/ = subject heading

**Database(s)**	**Search terms**
MEDLINE<br>Global Health	1. ebola.mp. OR (ebola adj1 virus).mp. OR Ebola haemorrhagic fever/ OR ebolavirus/ OR Zika fever/ OR zika.mp. OR (zika adj1 virus).mp. OR Zika Virus Infection/<br>2. financ*.mp. OR fund*.mp.OR funding/ OR finance/ OR spend*.mp. OR expenditure.mp. OR grant*.mp. OR loan*.mp.<br>3. Foundations/ OR "Organisation for Economic Co-Operation and Development"/ OR Red Cross/ OR United Nations/ OR unesco/ OR World Health Organization/ OR Pan American Health Organization/ OR global fund.mp. OR World Bank.mp. OR NGO*.mp. OR non-governmental organi?ation*.mp. OR non-government organi?ation*.mp. OR national government*.mp. OR government*.mp. OR IMF.mp. OR International monetary fund.mp. OR Global Health Security Agenda.mp. OR World Health Organi?ation.mp. OR International Working Group on Financing Preparedness.mp. OR United Nations.mp. OR charit*.mp.<br>4. 1 AND 2 AND 3
EMBASE	1. ebola.mp. OR (ebola adj1 virus).mp. OR Ebola haemorrhagic fever/ OR ebolavirus/ OR Zika fever/ OR zika.mp. OR (zika adj1 virus).mp. OR Zika Virus Infection/<br>2. financ*.mp. OR fund*.mp.OR funding/ OR finance/ OR spend*.mp. OR expenditure.mp. OR grant*.mp. OR loan*.mp. OR health care financing/<br>3. Foundations/ OR "Organisation for Economic Co-Operation and Development"/ OR Red Cross/ OR United Nations/ OR unesco/ OR World Health Organization/ OR Pan American Health Organization/ OR global fund.mp. OR World Bank.mp. OR NGO*.mp. OR non-governmental organi?ation*.mp. OR non-government organi?ation*.mp. OR national government*.mp. OR government*.mp. OR IMF.mp. OR International monetary fund.mp. OR Global Health Security Agenda.mp. OR World Health Organi?ation.mp. OR International Working Group on Financing Preparedness.mp. OR United Nations.mp. OR charit*.mp.<br>4. 1 AND 2 AND 3
Health Management Information Consortium (HMIC)	1. ebola.mp. OR (ebola adj1 virus).mp. OR Ebola haemorrhagic fever/ OR ebolavirus/ OR Zika fever/ OR zika.mp. OR (zika adj1 virus).mp. OR Zika Virus Infection/<br>2. financ*.mp. OR fund*.mp.OR funding/ OR finance/ OR spend*.mp. OR expenditure.mp. OR grant*.mp. OR loan*.mp.<br>5. 1 AND 2

Scopus	1. ebola* OR ebola AND virus OR ebola AND infection* OR ebola AND haemorrhagic AND fever* OR ebola AND hemorrhagic AND fever* OR zika* OR zika AND virus OR zika AND infection*<br>2. fund* OR financ* OR expenditure OR spend* OR grant* OR loan*<br>3. united AND nations OR un OR world AND health AND organisation OR world AND health AND organization OR non-government AND organisation OR non-governmental AND organisation OR non-government AND organization OR non-governmental AND organization OR charit* OR world AND bank OR imf OR international AND monetary AND fund OR global AND health AND security AND agenda OR foundation* OR ghsa OR global AND fund<br>4. 1 AND 2 AND 3
Web of Science	1. ebola OR (ebola AND virus*) OR (ebola AND infection*) OR (ebola AND haemorrhagic AND fever*) OR (ebola AND hemorrhagic AND fever) OR zika OR (zika AND virus*) OR (zika AND infection*)<br>2. financ* OR fund* OR expenditure OR spend* OR grant* OR loan*<br>3. (United AND Nations) OR (World AND Health AND Organisation) OR (World AND Health AND Organization) OR (non-government AND organisation) OR (non-governmental AND organisation) OR (non-government AND organization) OR (non-governmental AND organization) OR (Global AND Fund) OR (International AND Monetary AND Fund) OR IMF OR (World AND Bank) OR NGO* OR (Global AND Health AND Security AND Agenda) OR GHSA OR charit* OR foundation* OR government* OR (national AND government)<br>4. 1 AND 2 AND 3
EconLit	1. TX ((ebola OR ebola virus* OR ebola infection* OR ebola haemorrhagic fever* OR ebola hemorrhagic fever OR zika OR zika infection* OR zika virus*)<br>2. TX (financ* OR fund* OR expenditure OR loan* OR grant* OR spend*)<br>3. TX ((United AND Nations) OR (World AND Health AND Organisation) OR (World AND Health AND Organization) OR (non-government AND organisation) OR (non-governmental AND organisation) OR (non-government AND organization) OR (non-governmental AND organization) OR (Global AND Fund) OR (International AND Monetary AND Fund) OR IMF OR (World AND Bank) OR NGO* OR (Global AND Health AND Security AND Agenda) OR GHSA OR charit* OR foundation* OR government* OR (national AND government))<br>4. 1 AND 2 AND 3

Table A4. Inclusion and exclusion criteria for the literature review.

<table>
<tr><th>Inclusion</th><th>Exclusion</th></tr>
<tr><td rowspan="4">Related to Ebola and /or Zika from 2014 - 2019

Contains financial amounts donated or received by any actor

Funding for measures to respond to, prepare for or assist in the recovery for the outbreaks</td><td>Publishing date, study focus period or data range are pre-2014</td></tr>
<tr><td>Non-English</td></tr>
<tr><td>Not Open Access or accessible through Imperial College London Library</td></tr>
<tr><td>- Predictive modelling
- Estimations of economic losses due to the outbreaks
- Calls for funding or recommendations for future action or policy
- Estimations of funding need (e.g. vaccine)
- Funding requests to congress
- Total funding raised by a charity campaign*
- Ebola/Zika funding combined with funding for another disease
- In-kind contributions with no corresponding monetary value</td></tr>
</table>

*excluded as donations collected from the public are not necessarily equal to funding given to the implementing actor

Supplement 2 - Literature data sources

Literature by donor dataset

Table B1. Ebola donor pledged* amounts and data sources.

Donor	Amount pledged 2019 USD	Data source
African Development Bank	$248,727,200 - $411,267,200	(1,2)
African Union	$1,083,600 - $2,048,490	(2,3)
AU Staff Association	$107,210	(2)
Australia	$32,173,721 - $47,344,121	(1,2)
Austria	$3,269,905	(2)
Belgium	$60,198,415	(2)
Benin	$428,840	(2)
Bill and Melinda Gates Foundation	$62,567,756 - $116,747,756	(1,2)
Bolivia	$1,072,100	(2)
Brazil	$13,068,899	(2)
Burkina Faso	$128,652	(2)
Canada	$103,725,675 - $250,011,675	(1,2,4)
Central Emergency Response Fund	$16,254,000	(1)
Chad	$21,442	(2)
Children's Investment Fund Foundation	$21,672,000 - $43,114,000	(1,2)
Chile	$321,630	(2)
China	$134,334,130 - $332,632,930	(1,2,5)
Colombia	$107,210	(2)
Cote d'Ivoire	$1,072,100	(2)
Cyprus	$10,721	(2)
Czech Republic	$225,141	(2)
Denmark	$32,313,094	(2)
Estonia	$53,605	(2)
Ethiopia	$321,630	(2)
European Union	$1,007,055,693 - $1,348,124,893	(1-4)
Finland	$12,168,335 - $23,004,335	(1,2)
France	$203,270,160 - $351,723,360	(2,4)
Gambia	$536,050	(2)
Georgia	$32,163	(2)
Germany	$419,362,636 - $734,690,236	(1,2,4)
Google/Larry Page Family Foundation	$26,802,500	(2)
Guyana	$53,605	(2)
India	$14,086,800 - $24,807,800	(1,2)
International Monetary Fund	$461,003,000 - $921,930,953	(2,6)

Ireland	$40,450,333	(2)
Islamic Development Bank	$637,899,500	(2)
Israel	$9,380,875	(2)
Italy	$35,979,676	(2)
Japan	$198,188,406 - $245,866,806	(1,2)
Kazakhstan	$375,235	(2)
Kenya	$1,072,100	(2)
Latvia	$53,605	(2)
Luxembourg	$3,034,043	(2)
Malaysia	$107,210	(2)
Mali	$214,420	(2)
Malta	$64,326	(2)
Mark Zuckerberg and Pricilla Chan	$27,090,000	(1)
Mauritania	$321,630	(2)
Mauritius	$21,442	(2)
Mexico	$1,072,100	(2)
Montenegro	$10,721	(2)
Namibia	$1,072,100	(2)
Netherlands	$8,544,739 - $182,817,939	(1,2,4)
New Zealand	$2,648,087	(2)
Niger	$182,257	(2)
Nigeria	$4,824,450	(2)
Norway	$61,645,750	(2)
Paul G. Allen Family Foundation	$108,360,000 - $307,366,055	(1,2,4)
Phillippines	$2,176,363	(2)
Portugal	$32,163	(2)
Republic of Korea	$18,868,960	(2)
Romania	$42,884	(2)
Royal Charity Organisation of Bahrain	$1,072,100	(2)
Russia	$21,442,000	(2)
Saudi Arabia	$37,523,500	(2)
Senegal	$1,072,100	(2)
Sierra Leone	$18,690,000	(7)
Silicon Valley Community Foundation	$26,802,500	(2)
Spain	$14,677,049	(2)
Sweden	$84,520,800 - $174,417,310	(1,2,4)
Switzerland	$35,454,347	(2)
Togo	$2,144	(2)
Turkey	$1,929,780	(2)
United Kingdom	$1,072,368,025 - $1,632,589,225	(1,2,4)
UN Foundation	$139,373	(2)

US Biomedical Advanced Research and Development Authority (BARDA)	$45,836,280	(8)
United States	$2,534,551,610 - $11,528,431,610	(1,2,4,8,9)
Venezuela	$5,360,500	(2)
Volvo Group	$1,404,451	(2)
Wellcome Trust	$5,634,720	(10)
West African Economic and Monetary Union	$4,824,450	(2)
World Bank	$2,034,657,800 - $2,934,045,800	(1,2,4,11,12)

**Pledge = announcement of funding intent*

Table B2. Ebola donor commitments* and data sources.

Donor	Amount committed 2019 USD	Data source
European Union	$1,321,400,000	(13)

**Commitment = agreed funding obligation, usually in writing*

Table B3. Ebola donor disbursements* and data sources.

Donor	Amount disbursed 2019 USD	Data source
African Development Bank	$120,539,150	(2,14)
African Union	$964,890	(2)
AU Staff Association	$107,210	(2)
Australia	$32,573,721	(2,14)
Austria	$3,269,905	(2)
Belgium	$55,459,733	(2)
Benin	$428,840	(2)
Bill and Melinda Gates Foundation	$62,231,645	(2,14)
Bolivia	$1,072,100	(2)
Brazil	$13,068,899	(2)
Burkina Faso	$128,652	(2)
Canada	$194,116,621 - $278,637,421	(2,4,14,15)
CDC's Epidemiology and Laboratory for Infectious Diseases (ELC)	$107,210,000	(16)
Chad	$0	(2)
Children's Investment Fund Foundation	$21,442,000	(2)
Chile	$321,630	(2)
China	$144,068,280 - $194,997,480	(2,5,14)
Colombia	$107,210	(2)
Cote d'Ivoire	$0	(2)
Cyprus	$10,721	(2)

Czech Republic	$225,141	(2)
Denmark	$37,113,094	(2,14)
Estonia	$53,605	(2)
Ethiopia	$321,630	(2)
European Union	$819,916,390 - $901,186,390	(2,4,14)
Finland	$12,168,335	(2)
France	$118,028,800 - $221,593,660	(2,4,14)
Gambia	$536,050	(2)
Georgia	$32,163	(2)
Germany	$182,276,000 - $326,248,309	(2,4,14)
Global Alliance for Vaccines and Immunisation (GAVI)	$26,500,000	(14)
Google/Larry Page Family Foundation	$0	(2)
Guyana	$53,605	(2)
India	$10,721,000	(2)
International Monetary Fund	$460,927,953 - $870,528,753	(2,17)
Ireland	$43,850,333	(2,14)
Islamic Development Bank	$13,755,043	(2)
Israel	$9,380,875	(2)
Italy	$14,151,156	(2,14)
Japan	$203,288,406	(2,14)
Kazakhstan	$375,235	(2)
Kenya	$0	(2)
Latvia	$53,605	(2)
Luxembourg	$3,634,043	(2,14)
Malaysia	$107,210	(2)
Mali	$214,420	(2)
Malta	$64,326	(2)
Mauritania	$321,630	(2)
Mauritius	$21,442	(2)
Mexico	$1,072,100	(2)
Montenegro	$10,721	(2)
Namibia	$0	(2)
Netherlands	$85,768,000 - $150,784,000	(2,4)
New Zealand	$2,648,087	(2)
NGOs	$8,000,000	(18)
Niger	$182,257	(2)
Nigeria	$12,678,120 - $17,502,570	(2,3)(2,3)
Norway	$65,145,750	(2,14)
Paul G. Allen Family Foundation	$76,551,075 - $136,149,075	(2,4,14)
Philippines	$2,176,363	(2)
Portugal	$32,163	(2)
Republic of Korea	$18,868,960	(2)
Romania	$42,884	(2)

Royal Charity Organisation of Bahrain	$1,072,100	(2)
Russia	$21,442,000	(2)
Saudi Arabia	$0	(2)
Senegal	$0	(2)
Silicon Valley Community Foundation	$0	(2)
South Korea	$1,000,000	(14)
Spain	$13,401,250	(2)
Susan T.Buffet Foundation	$5,000,000	(14)
Sweden	$87,637,200 - $97,178,890	(2,4,14)
Switzerland	$36,854,347	(2,14)
Togo	$2,144	(2)
Turkey	$321,630	(2)
UK	$632,407,675 - $959,654,875	(2,4,14)
UN Central Emergency Response Fund (CERF)	$12,600,000	(14)
UN Foundation	$139,373	(2)
UN OCHA/DRC Humanitarian Fund	$10,000,000	(14)
UNICEF	$300,000	(14)
US Biomedical Advanced Research and Development Authority (BARDA)	$26,981,640	(19)
United States	$2,786,951,610 - $6,951,197,170	(2,4,8,9,14,18,20)
Venezuela	$5,360,500	(2)
Volvo Group	$1,404,451	(2)
Wellcome Trust	$4,200,000	(14)
West African Economic and Monetary Union	$4,824,450	(2)
WHO Contingency Fund for Emergencies (CFE)	$75,135,600	(14,21)
World Bank	$573,101,776 - $699,882,976	(2,4)
World Bank International Development Association (IDA)	$120,000,000	(14)
World Bank Pandemic Emerency Financing Facility (PEF)	$50,000,000	(14)
World Food Programme	$500,000	(14)

**Disbursement = an actual transfer of money*

Table B4. Zika donor pledged* amounts and data sources.

Donor	Amount pledged 2019 USD	Data source
Brazil	$259,426,500	(22)
European Union	$29,992,000 - $45,687,400	(23,24)
Governments	$29,257,200	(25)
United Kingdom	$7,953,750	(23)
US Department of Health and Human Services	$85,900,500	(26)
United States	$371,175,000	(16)
World Health Organization	$59,388,000	(27)

**Pledge = announcement of funding intent*

Table B5. Zika donor disbursements and data sources.

Donor	Amount disbursed 2019 USD	Data source
Brazil	$59,081,670	(22)
United Kingdom	$6,938,685	(28)
US Centers for Disease Control and Prevention (CDC)	$41,359,500	(29)
United States	$1,781,640,000	(23,26,29)

**Disbursement = an actual transfer of money*

Literature by recipient dataset

Table B6. Ebola pledges* received and data sources.

Recipient	Amount received in pledges 2019 USD	Data source
ACDI/VOCA	$19,297,800	(2)
African Union	$22,996,545	(2)
American Refugee Committee	$8,180,123	(2)
Benin	$557,492	(2)
Burkina Faso	$21,442	(2)
Burundi	$21,442	(2)
Cameroon	$428,840	(2)
Catholic Relief Services	$15,084,447	(2)
Concern	$7,301,001	(2)
Cote D'Ivoire	$63,789,950	(2)
Ebola MPTF	$144,797,826	(2)
FAO	$8,265,891	(2)
Gabon	$96,489	(2)
Ghana	$6,797,114	(2)
Global Communities	$36,494,284	(2)
GOAL	$17,368,020	(2)

Guinea	$895,407,199	(2)
IFRC	$120,965,043	(2)
International Medical Corps	$71,241,045	(2)
International Rescue Committee	$45,703,623	(2)
IOM	$76,001,169	(2)
Jhpiego	$9,327,270	(2)
Liberia	$716,259,289	(2)
Mali	$3,398,557	(2)
Mapp Pharmaceuticals	$45,836,280	(8)
Medicine San Frontieres	$20,938,113	(2)
Mercy Corps	$35,347,137	(2)
Netherlands Ministry of Defence	$7,772,725	(2)
Niger	$21,442	(2)
Nigeria	$32,163	(2)
Not specified	$2,422,206,251	(2)
Other government institutions	$38,381,180	(2)
Other NGO	$466,020,428	(2)
Other other	$72,205,935	(2)
Partners in Health	$37,705,757	(2)
Project Concern International	$20,788,019	(2)
Research Institutions (Various)	$235,937,047	(2)
Samaritan's Purse	$10,431,533	(2)
Save the Children	$33,803,313	(2)
Senegal	$4,910,218	(2)
Sierra Leone	$816,822,269	(2)
Swedish Civil Contingencies Agency	$1,543,824	(2)
UN OCHA	$7,526,142	(2)
UN Women	$1,093,542	(2)
UNDP	$28,056,857	(2)
UNFPA	$20,337,737	(2)
UNHCR	$7,354,606	(2)
UNICEF	$335,245,670	(2)
UNOPS	$58,118,541	(2)
Unspecified government	$224,294,041	(2)
US Assistant Secretary for Preparedness and Response (ASPR)	$954,169,000	(30)
US Biomedical Advanced Research and Development Authority (BARDA)	$62,181,800	(30)
US Centers for Disease Control and Prevention (CDC)	$1,929,780,000 - $4,733,478,136	(2,16,30)

US Centres for Disease Control Foundation	$19,287,079	(2)
US Department of Defence	$797,385,096	(2,30)
US Food and Drug Administration (FDA)	$26,802,500	(30)
US National Institute of Health (NIH)	$255,159,800	(30)
US State Department and USAID	$2,787,460,000	(30)
WFP	$323,956,457	(2)
WHO	$381,710,484	(2)
World Bank	$53,658,605	(2)
World Food Programme (WFP)	$6,501,600	(5)
World Vision	$7,086,581	(2)

**Pledge = announcement of funding intent*

Table B7. Ebola disbursements* received and data sources.

Recipient	Amount received 2019 USD	Data source
ACDI/VOCA	$19,297,800	(2)
African Union	$20,530,715	(2)
American Refugee Committee	$8,180,123	(2)
Benin	$557,492	(2)
Burkina Faso	$21,442	(2)
Burundi	$21,442	(2)
Cameroon	$428,840	(2)
Catholic Relief Services	$15,084,447	(2)
Concern	$7,301,001	(2)
Cote D'Ivoire	$63,789,950	(2)
Ebola MPTF	$138,440,273 - $144,941,873	(2,5)
FAO	$6,657,741	(2)
Gabon	$96,489	(2)
Ghana	$19,791,151	(2,4)
Global Communities	$36,494,284	(2)
GOAL	$17,368,020	(2)
Guinea	$364,278,138 - $616,811,118	(2,4,5)
IFRC	$123,859,713	(2)
International Medical Corps	$69,954,525	(2)
International Rescue Committee	$45,703,623	(2)
IOM	$76,001,169	(2)
Ivory Coast	$23,839,200	(4)
Jhpiego	$9,327,270	(2)

Liberia	$955,735,200 - $1,506,151,340	(2,4)
Mali	$15,178,784	(2,4)
Mapp Pharmaceuticals	$26,981,640	(19)
Medicine San Frontieres	$19,222,753	(2)
Mercy Corps	$35,347,137	(2)
Netherlands Ministry of Defence	$7,772,725	(2)
Niger	$21,442	(2)
Nigeria	$17,012,520 - $17,044,683	(2-4)
Not specified	$678,864,441	(2)
Other government institutions	$19,490,778	(2)
Other NGO	$442,455,670	(2)
Other other	$61,313,399	(2)
Partners in Health	$37,705,757	(2)
Project Concern International	$20,788,019	(2)
Research Institutions (Various)	$291,000,103	(2)
Samaritan's Purse	$10,431,533	(2)
Save the Children	$33,267,263	(2)
Senegal	$7,517,758	(2,4)
Sierra Leone	$501,706,800 - $951,388,424	(2,4)
Swedish Civil Contingencies Agency	$1,543,824	(2)
UN OCHA	$6,443,321	(2)
UN Women	$1,093,542	(2)
UNDP	$28,056,857	(2)
UNFPA	$20,337,737	(2)
UNHCR	$7,354,606	(2)
UNICEF	$335,245,670	(2)
United Nations	$1,500,940,000	(31)
UNOPS	$44,867,385	(2)
Unspecified government	$7,912,098	(2)
US Centers for Disease Control and Prevention (CDC)	$1,917,972,000 - $2,771,535,136	(2,9)
US Centres for Disease Control Foundation	$18,214,979	(2)
US Department of Defence	$677,309,896	(2)
US jurisdictions	$107,210,000	(16)
World Food Programme	$322,359,028	(2)
WHO	$436,891,471 - $444,476,671	(2,4,15)
World Bank	$53,658,605	(2)
World Vision	$7,086,581	(2)

**Disbursement = an actual transfer of money*

Table B8. Zika pledges* received and data sources.

Recipient	Amount received in pledges 2019 USD	Data source
Brazil Armed Forces	$37,443,000	(22)
Brazil Ministry of Health (MoH)	$160,470,000	(22)
Brazil	$61,513,500	(22)

**Pledge = announcement of funding intent*

Table B9. Zika disbursements* received and data sources.

Recipient	Amount received 2019 USD	Data source
Excivion Ltd	$759,700	(28)
Jenner Institute, Oxford University	$757,983	(28)
Prokarium Ltd	$599,262	(28)
Puerto Rico Science, Technology, and Research Trust	$14,847,000	(29)
Stabilitech Limited	$325,562	(28)
Themis Ltd	$1,518,154	(28)
US Centers for Disease Control and Prevention (CDC)	$371,175,000	(29)
US jurisdictions	$26,512,500	(29)

**Disbursement = an actual transfer of money*

Table B10. Ebola disbursements by donor for each actor type.

Type	Amount disbursed 2019 USD
Government	$4,855,622,177 – $9,809,484,246
Multilateral	$2,289,311,835 – $£2,906,963,835
National agency	$134,191,640
Private	$170,829,171 – $230,427,171
NGO	$9,072,100

**Disbursement = an actual transfer of money*

Literature data source references

(1) Glassman A, Raghavan S. How Much Is Actually Being Spent on Ebola? Center For Global Development. Weblog. Available from: https://www.cgdev.org/blog/how-much-actually-being-spent-ebola [Accessed 27th April 2020].

(2) Office of the United Nations Special Envoy on Ebola. Resources for Results V. United Nations. Report number: 5, 2016.

(3) Waheed Y. Ebola in West Africa: an international medical emergency. *Asian Pacific Journal of Tropical Biomedicine.* 2014;4(9):673-674. Available from: doi:10.12980/APJTB.4.201414B389

(4) Grépin KA. International donations to the Ebola virus outbreak: too little, too late? *BMJ.* 2015;350:h376. Available from: doi:10.1136/bmj.h376

(5) Huang Y. China's Response to the 2014 Ebola Outbreak in West Africa. *Global Challenges.* 2017;1(2):1600001. Available from: doi:10.1002/gch2.201600001

(6) Kentikelenis A, King L, McKee M, Stuckler D. The International Monetary Fund and the Ebola outbreak. *The Lancet Global Health.* 2015;3(2):e69-e70. Available from: doi:10.1016/S2214-109X(14)70377-8

(7) O'Carroll L. A third of Sierra Leone's Ebola budget unaccounted for, says report. *The Guardian.* February 16, 2015. Available from: http://www.theguardian.com/world/2015/feb/16/ebola-sierra-leone-budget-report [Accessed 5th May 2020].

(8) Nature Biotechnology. Outpaced by an outbreak. *Nature Biotechnology.* 2014;32(11):1067-1067. Available from: doi:10.1038/nbt.3074

(9) Standley CJ, MacDonald PDM, Attal-Juncqua A, Barry AM, Bile EC, Collins DL, et al. Leveraging Partnerships to Maximize Global Health Security Improvements in Guinea, 2015-2019. *Health Security.* 2020;18(S1):S34-S42. Available from: doi:10.1089/hs.2019.0089

(10) Gulland A. Clinical trials of Ebola treatment to start in Africa. *BMJ.* 2014;349(g5838) Available from: doi:10.1136/bmj.g5838

(11) Green A. WHO and partners launch Ebola response plan. *The Lancet.* 2014;384(9942): 481. Available from: doi:10.1016/S0140-6736(14)61322-2

(12) Jonas O. Pandemic bonds: designed to fail in Ebola. *Nature.* 2019;572(7769):285-285. Available from: doi:10.1038/d41586-019-02415-9

(13) Galpin C. Chronology: The European Union in 2014. *JCMS: Journal of Common Market Studies.* 2015;53(S1):230-236. Available from: doi:10.1111/jcms.12258

(14) Moss K, Michaud J, Kates J. Data Note: Donor Funding for the Current Ebola Response in the DRC. *KFF: Global Health Policy.* Weblog. Available from: https://www.kff.org/global-health-policy/issue-brief/data-note-donor-funding-for-the-current-ebola-response-in-the-drc/ [Accessed 27th April 2020].

(15) Plummer FA, Jones SM. The story of Canada's Ebola vaccine. *CMAJ.* 2017;189(43):E1326-E1327. Available from: doi:10.1503/cmaj.170704

(16) Fischer LS, Santibanez S, Jones G, Anderson B, Merlin T. How Is CDC Funded to Respond to Public Health Emergencies? Federal Appropriations and Budget Execution Process for Non–Financial Experts. *Health Security.* 2017;15(3):307-311. Available from: doi:10.1089/hs.2017.0009

(17) Cangul M, Sdralevich C, Sian I. Beating Back Ebola. *Finance & Development.* June 2017. Available from: https://www.imf.org/external/pubs/ft/fandd/2017/06/cangul.htm [Accessed 5th May 2020].

(18) Nature. To contain Ebola, the United States must fulfil its promise to the World Health Organization. *Nature.* 2019; 571 (7764): 145-145. Available from: doi:10.1038/d41586-019-02118-1

(19) McCarthy M. US signs contract with ZMapp maker to accelerate development of the Ebola drug . *BMJ.* 2014; 349 (g5488): Available from: doi:10.1136/bmj.g5488

(20) Glassman A. After Ebola*. Finance & Development.* June 2016. Available from: https://www.imf.org/external/pubs/ft/fandd/2016/06/glassman.htm [Accessed 27th April 2020].

(21) Yaqub F. 2018: a year in review. *The Lancet.* 2018;392(10165):2669-2670. Available from: doi: 10.1016/S0140-6736(18)33244-6

(22) Gómez EJ, Perez FA, Ventura D. What explains the lacklustre response to Zika in Brazil? Exploring institutional, economic and health system context. *BMJ Global Health.* 2018;3(5):e000862. Available from: https://gh.bmj.com/content/3/5/e000862 [Accessed 27th April 2020]

(23) Shuchman M. Canada's support lags for research on Zika. *CMAJ : Canadian Medical Association Journal.* 2016;188(10):E203-E204. Available from: doi:10.1503/cmaj.109-5284

(24) Goodridge K, Reveiz L, Elias V. An overview of financial sources being utilized to support Zika Virus published research. *PLoS ONE.* 2017;12(8):e0183134. Available from: doi:10.1371/journal.pone.0183134

(25) Gostin LO, Hodge JG. Is the United States Prepared for a Major Zika Virus Outbreak? *JAMA.* 2016;315(22):2395-2396. Available from: doi:10.1001/jama.2016.4919

(26) Morabito KM, Graham BS. Zika Virus Vaccine Development. *The Journal of Infectious Diseases.* 2017;216(suppl_10):S957-S963. Available from: doi:10.1093/infdis/jix464

(27) McCarthy M. WHO sets out $56m Zika virus response plan. *BMJ.* 2016;352(i1042) Available from: doi:10.1136/bmj.i1042

(28) UK Department of Health and Social Care. *UK Zika research awarded share of £120 million vaccine fund.* Available from: https://www.gov.uk/government/news/uk-zika-research-awarded-share-of-120-million-vaccine-fund [Accessed 5th May 2020].

(29) Murthy BP, Vagi S, Desamu-Thorpe R, Avchen R. *Morbidity and Mortality Weekly Report: Assessment of State, Local, and Territorial Zika Planning and Preparedness Activities — United States, June 2016–July 2017.* US Centers for Disease Control and Prevention. Report number: 67, 2018.

(30) Boddie C, Sell TK, Watson M. Federal Funding for Health Security in FY2016. *Health Security.* 2015;13(3):186-206. Available from: doi:10.1089/hs.2015.0017

(31) Standley CJ, Sorrell EM, Kornblet SA, Fischer JE, Katz R. Linking funds to actions for global health emergencies. *Science.* 2015; 348 (6236): 762-763. Available from: doi:10.1126/science.aaa5521

Supplement 3 – Results

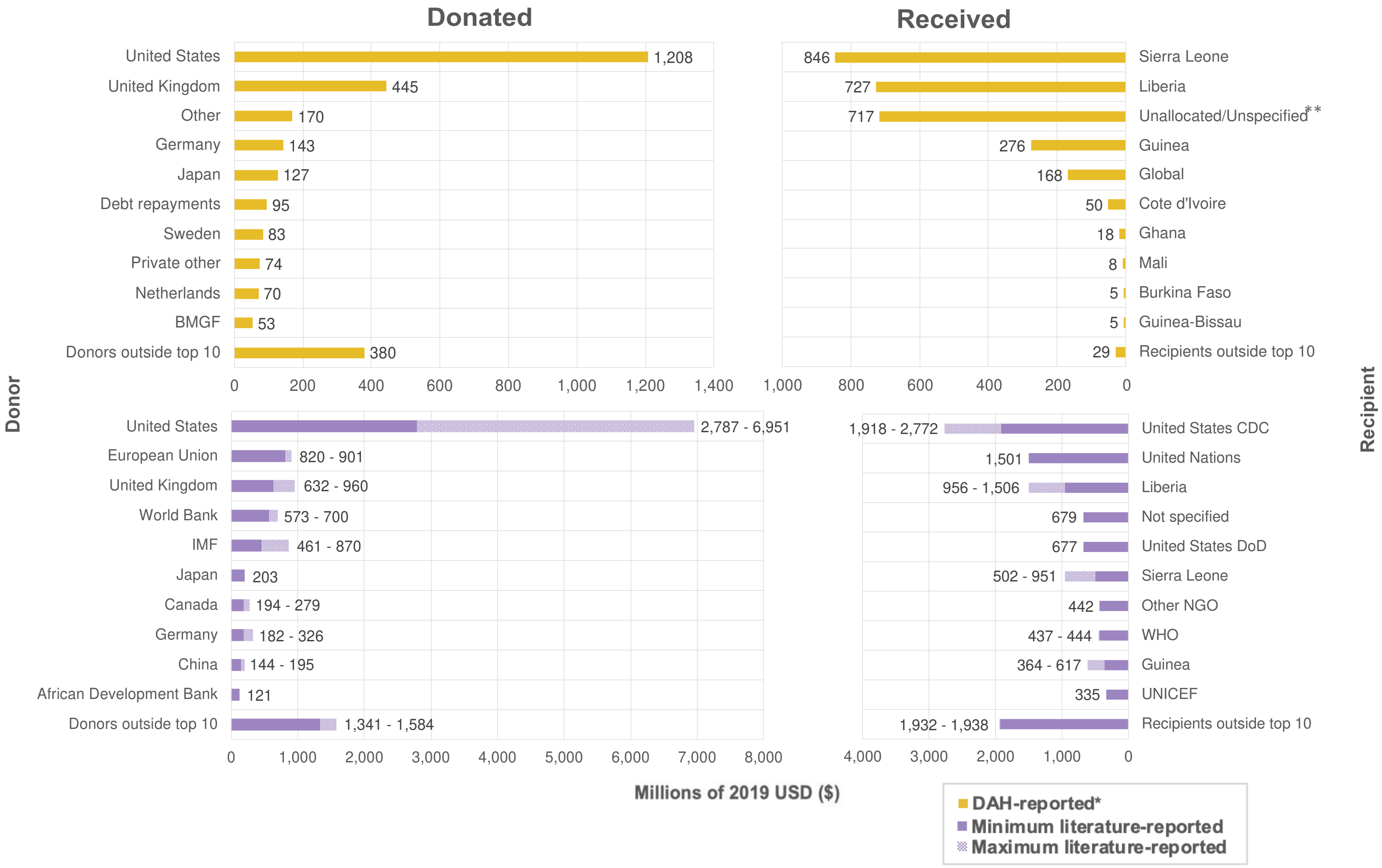

Figure C1. Totals disbursed by the top 10 donors and received by the top 10 recipients according to the DAH database and the literature for Ebola.

DAH = Development Assistance for Health; BMGF = Bill and Melinda Gates Foundation; NGO = Non-governmental organisation; IMF = International Monetary Fund; CDC = Centers for Disease Control and Prevention; DoD = Department of Defence; WHO = World Health Organization; UNICEF = United Nations Children's Fund. Other = interest, transfer of funds, refunds and miscellaneous income.

**DAH data only available 2014-2018*

***The OECD CRS defines unspecified funding as" non-country programmable aid such as administrative costs, refugees in donor country and research costs" and unallocated funding as funding that "cannot be classified in any of the other non-country programmable aid categories[1]. It is not clear whether these definitions are applicable to all DAH data.*

[1] OECD. *Country programmable aid (CPA): Frequently asked questions*. Available from: https://www.oecd.org/development/effectiveness/countryprogrammableaidcpafrequentlyaskedquestions.htm [Accessed 28th February 2021]

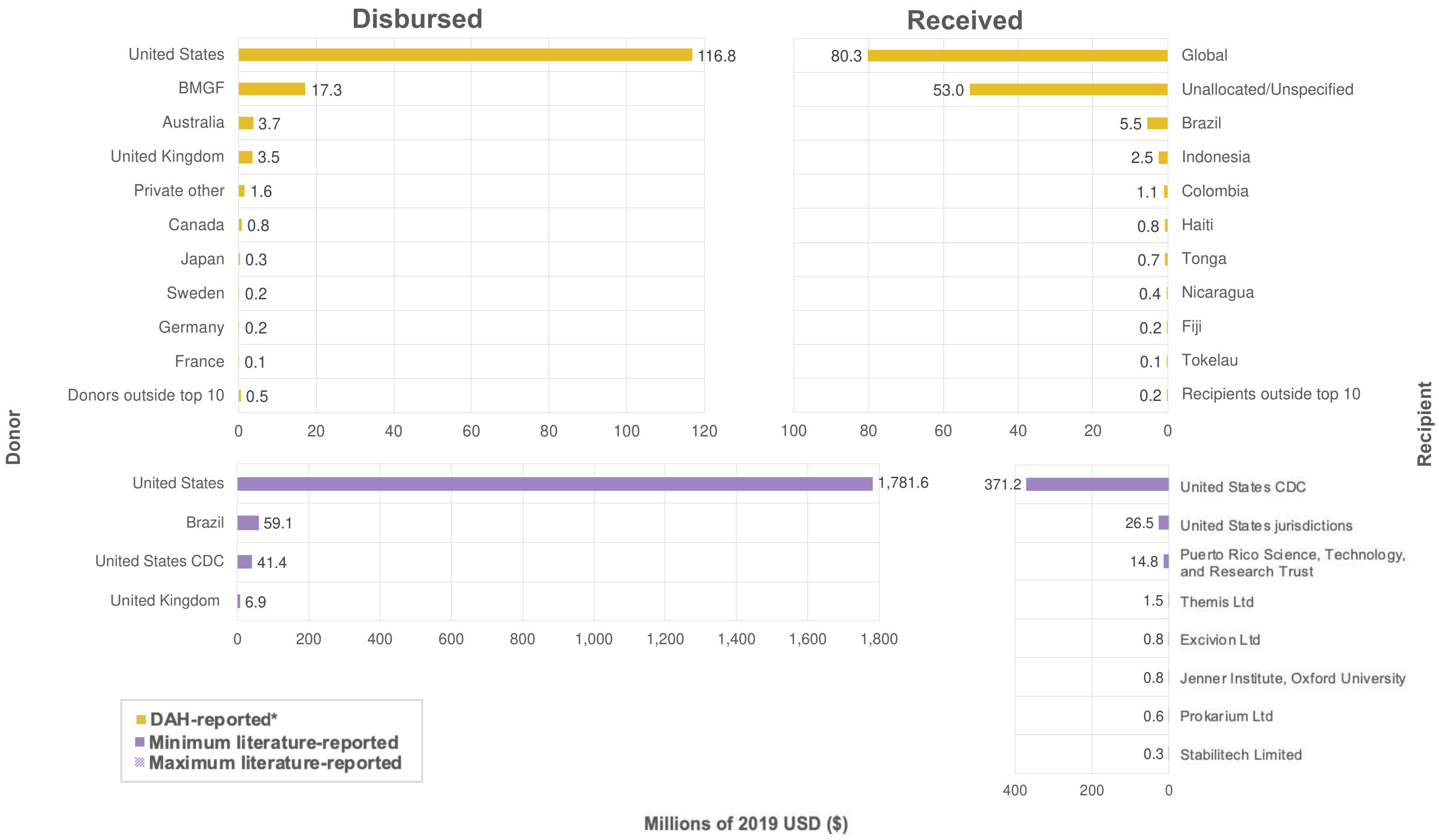

Figure C2. Total disbursed by the top 10 donors and received by the top 10 recipients according to the DAH database and the literature for Zika.

DAH=Development Assistance for Health; BMGF = Bill and Melinda Gates Foundation; CDC = Centers for Disease Control and Prevention

**DAH data only available 2014-2018*

***The OECD CRS defines unspecified funding as" non-country programmable aid such as administrative costs, refugees in donor country and research costs" and unallocated funding as funding that "cannot be classified in any of the other non-country programmable aid categories[1]. It is not clear whether these definitions are applicable to all DAH data.*

[1] OECD. *Country programmable aid (CPA): Frequently asked questions.* Available from: https://www.oecd.org/development/effectiveness/countryprogrammableaidcpafrequentlyaskedquestions.htm [Accessed 28th February 2021]

Table C1. Total amount disbursed, funded and committed, and pledged to Ebola, as calculated by existing published articles.

UN = United Nations; OCHA = Office for the Coordination of Humanitarian Affairs; MPTF = Multi-Partner Trust Fund; DRC = Democratic Republic of the Congo; WHO = World Health Organization; USAID = United States Agency for International Development; CERF = Central Emergency Response Fund

**Only the top 20 donors **OCHA amounts include commitments and disbursements*

**2014 – 2015 Ebola outbreak**				
**First author**	**Year published**	**Data source(s)**	**Dates**	**Amount 2019 USD**
**Disbursements**				
Grépin[24]	2015	UN OCHA FTS	Up until 31/12/2014	$1,083,600,000
Office of the United Nations Special Envoy on Ebola[23]	2016	Information requests; Ebola MPTF	Sept 2014 - Jan 2015	$6,325,390,000
**Funded and committed combined**				
Glassman*[20]	2014	UN OCHA FTS	Jan 2014 - Oct 2014	$1,517,040,000
**Pledges**				
Glassman[22]	2016	UN OCHA	2014 - 2015	$3,881,002,000
Grépin[24]	2015	UN OCHA FTS	Up until 31/12/2014	$3,131,604,000
Glassman*[20]	2014	Press releases	Jan 2014 - Oct 2014	$2,600,640,000
Office of the United Nations Special Envoy on Ebola[23]	2016	Information requests; Ebola MPTF	Sept 2014 - Jan 2015	$9,541,690,000

**2018 – 2019 Democratic Republic of Congo Ebola outbreak**				
**First author**	**Year published**	**Data source**	**Dates**	**Amount 2019 USD**
**Disbursements**				
Moss[25]	2019	DRC/Partners SRP 3; WHO; OCHA**; European Union; UK; USAID; Gavi; DRC/Partners SRP 2; U.N. CERF; World Bank	Aug 2018 - Dec 2019	$734,000,000
